# *Gymnema sylvestre* Extract Restores the Autophagic Pathway in Human Glioblastoma Cells U87Mg

**DOI:** 10.3390/biology10090870

**Published:** 2021-09-04

**Authors:** Rossella Rotondo, Salvatore Castaldo, Maria Antonietta Oliva, Antonietta Arcella

**Affiliations:** I.R.C.C.S I.N.M. Neuromed, Via Atinense, 18, 86077 Pozzilli, IS, Italy; rossellaross1988@gmail.com (R.R.); salvatore.castaldo@neuromed.it (S.C.); mariaantonietta.oliva@neuromed.it (M.A.O.)

**Keywords:** glioblastoma, *Gymnema sylvestre* (GS), autophagy, mTOR

## Abstract

**Simple Summary:**

The treatment of GBM is extremely difficult and complicated by the heterogeneous nature of neoplastic cells. The problems inherent in treating any central nervous system tumour are due to the anatomical complexity and the limited repair mechanisms of the surrounding unaffected tissues. The choice of the most suitable treatment for GBM depends on several factors: the location of the disease, the extent, and the nature of the tumour. The limit of this choice is mainly due to the degree of complexity of the disease and to the mechanisms of drug resistance that the neoplasm develops during the treatment. Herbal medicines and their derived phytocompounds are increasingly recognised as useful complementary treatments for cancer. Numerous clinical studies have reported the beneficial effects of herbal medicines on survival, immune modulation, and quality of life of cancer patients when used in combination with conventional therapies. In this study, we investigated all the mechanisms that control tumour cell growth after induction with *Gymnema sylvestre* (GS) extract and the key proteins that regulate these mechanisms in glioblastoma cells. The study is of great translational interest because the natural substances used could be proposed as natural adjuvant drugs for the treatment of glioblastoma, and therefore could act by modulating new molecular targets for the control of brain tumour cell growth.

**Abstract:**

Glioblastoma is a brain tumour, characterised by recurrent or innate resistance to conventional chemoradiotherapy. Novel natural molecules and phyto-extracts have been proposed as adjuvants to sensitise the response to Temozolomide (TMZ). In this study, we investigated the effect of GS extract on human glioblastoma cells U87Mg. According to the IC50-values, GS extract displayed a significant cytotoxicity. This was confirmed by cell growth inhibition and alteration in metabolic activity evaluated by cell count and MTT assay. GS induced reduction in Pro-caspase 9, 3, but not PARP cleavage nor DNA fragmentation. Thus, in GS-induced cytotoxicity, cell death is not associated with apoptosis. In this context, short-term treatment of U87Mg cells with GS extract (1 mg/mL) reduced the phosphorylation levels of mTOR and of its downstream target P70 S6 kinase, highlighting the role of GS extract into autophagy induction. The activation of autophagic flux by GS extract was confirmed by Western blot analysis, which revealed the reduction in p62 and the concomitant increase in LC3B II/I ratio. Immunofluorescence evidenced the accumulation of LC3B puncta in U87Mg cells pretreated with autophagy inhibitor Bafilomycin A1. Furthermore, as main key regulators of type II programmed cell death, p53, p21 and CDK4 were also investigated and were inhibited by GS treatment. In conclusion, GS extract could be considered as an autophagy inducer in glioblastoma cells U87Mg.

## 1. Introduction

Glioblastoma is the most severe and common brain tumour. It is a malignant and infiltrating tumour, characterised by expansive and rapid growth. These aspects, together with high angiogenesis, cellular heterogeneity, and the presence of cancer stem cells able to proliferate and generate neoplastic cells, contribute to a poor prognosis. The average survival of the patients with this type of cancer is 14.6 months [1], with a 5-year survival rate of 2% [2]. There are numerous histopathological variants of glioblastoma multiforme (GBM): in any case, the common characteristics are microvascular proliferation, cellular and nuclear pleomorphism, and necrosis. Furthermore, a high resistance to drug treatments characterises GBM. This resistance is attributable to the ability of GBM cells to activate different resistance mechanisms (cell defense factors, DNA repair) in response to chemotherapy and radiotherapy, complicating effective therapy for this tumour [3]. Current clinical practice is based on a standard therapeutic program involving surgical resection of the solid tumour volume and adjuvant radiochemotherapy, known as Stupp protocol [1]. In most cases, despite therapy, relapse occurs after a period of time, which varies from patient to patient. In the last decade, numerous investigations have been focused on the key proteins that alter the mechanisms of control of GBM homeostasis. Alterations of the cellular autophagy mechanism are implicated in the tumourigenesis of brain gliomas and in the response to radiochemotherapy treatment [4].

In GBM, the upregulation of molecular target of rapamycin (mTOR) has been associated with the cell growth rate, stem cell proliferation, and cancer relapse [5,6,7]. In our previous study, we demonstrated that rapamycin inhibits GBM cell growth, in vitro and in vivo [8].

Gliomas present a reduction in active autophagy linked to a more aggressive neoplastic phenotype [9,10]. In high-grade gliomas, low levels of Beclin 1 expression identified niches of patients with more aggressive malignancies (worst KPS and worst survival). Pre-clinical and clinical studies showed a key role of cellular autophagy in response to GBM treatment [11].

Many novel molecules from natural sources have been reported to exert anti-cancer effects through modulation of autophagy pathway [12].

GS R. Br. is a perennial woody climbing plant belonging to Apocynaceae family, commonly found in tropical and subtropical regions of India, China, Africa, Malaysia, and Sri Lanka. Ethanolic GS extract is a potent antidiabetic drug used in traditional Ayurveda medicine. The anticancer activity of GS extract was evaluated in different human cancer cell lines [13,14]. The aim of this study was to investigate the involvement of GS in the growth control of glioblastoma cells U87Mg and the cell death mechanisms involved.

## 2. Materials and Methods

### 2.1. Cell Culture

Human continuous glioblastoma cell line U87Mg was purchased from Sigma Aldrich Collection (LGC Promochem, Teddington, UK). U87Mg cells were grown in Dulbecco’s Modified Eagle’s Medium supplemented with 10% fetal bovine serum, 2 mmol/L L-glutamine, 100 IU/mL penicillin, 100 μg/mL streptomycin at 37 °C, 5% CO_2_, and 95% humidity. For in vitro treatment, Native GS extract, United States Pharmacopeia (USP) Reference Standard was purchased from Merck (KGaA, Darmstadt, Germany).

### 2.2. Evaluation of Half-Maximal Inhibitory Concentration (IC50) of GS Extract

The IC50-values of GS at 24, 48, and 72 h on the human GBM continuous cell line U87Mg were estimated by plating 5 × 10^3^ cells/well in 96-well plates. The IC50-values were determined treating cells with GS extract 0.05, 0.1, 0.5, 1, and 3 mg/mL and DMSO 0.3% as vehicle control, respectively, and the IC50-values were assessed with GraphPad Prism software (GraphPad Software Inc., San Diego, CA, USA).

### 2.3. Treatment of Human GBM Cell Line U87Mg with GS Extract

To evaluate the effects of GS on cell growth, U87Mg cells were plated in 48-well plates at 1 × 10^4^ cells/well in DMEM supplemented with 10% FBS and incubated at 37 °C in an atmosphere containing 5% CO_2_. From the following day the cells were treated every 24 h with GS at concentrations of 0.5 and 1 mg/mL for 24, 48, and 72 h. DMSO 0.3% was used as vehicle control. After treatment, cell counts were performed every day using a Burker chamber.

### 2.4. Cell Viability Assay

The long-term effect of GS on cell viability was evaluated by seeding 5 × 10^3^ cells in 96-well plates and allowing them to adhere for 12 h. U87Mg cells were treated with GS 0.5 and 1 mg/mL every 24 h for 24, 48, and 72 h followed by MTT (3-(4,5-dimethylthiazol-2-yl)-2,5-diphenyltetrazolium bromide) (Sigma–Aldrich, St. Louis, MO, USA) assay. DMSO 0.3% was used as vehicle control. Briefly, 5 mg/mL MTT in 100 µL of DMEM was added to the cultured cells. Formazan crystals were dissolved in an acidic isopropanol solution and the absorbance of solution determined at 595 nm.

### 2.5. Clonogenic Assay

The clonogenic assay was performed seeding 10^3^ U87Mg cells/well in triplicate in a 6-well plate for 48 h. Cells were treated, respectively, with 1 mg/mL GS and DMSO 0.3% as vehicle control for 24 h, and the medium replaced every 3 days for 14 days. The colonies were fixed with 4% paraformaldehyde solution for 5 min, washed with PBS, and stained with crystal violet 0.05% for 30 min to be counted under the microscope.

### 2.6. Wound-Healing Assay

Cell motility was evaluated by seeding GBM cells into 6-multiwell culture plates. At 90% of confluence, a scratch was gently caused through the cell monolayer by sterile 100 μL pipette tips, and the detached cells were washed away. Cells were treated, respectively, with GS 1 mg/mL and DMSO 0.3% as vehicle control, and migration was imaged under an Evos FL microscope (Life Technologies, Thermo Fisher Scientific, CA, USA) for each time point (T0, T6 h, T24 h and T48 h).

### 2.7. Western Blot Analysis of U87Mg Cells Treated with GS Extract

Proteins were extracted from U87Mg cells treated for short and long-term treatment with GS extract (1 mg/mL GS) in Triton X-100 lysis buffer (Tris-HCL 10 mM, EDTA 1 mM, NaCl 150 mM, Triton X-100 1%, NaF 1 mM, Na_4_P_2_O_7_ 1 mM, Na_3_VO_4_ 1 mM, and protease inhibitors 1×). Proteins (15 μg) were separated by sodium dodecyl sulfate polyacrylamide gel electrophoresis (SDS-PAGE) and were transferred to PVDF membranes by electroblotting. The membranes were incubated for 1 h at room temperature in 5% milk or BSA diluted in 1× Tris-buffered saline containing Tween-20 (TBS-T) and then incubated overnight at 4 °C with primary antibodies specific for CDK4 (Cell Signaling Danvers, MA, USA; 1:1000), p21 (Cell Signaling, 1:1000), p53 (Roche, 1:1000), NF-κB p65 (Cell Signaling, 1:1000), SQSTM1/p62 (Cell Signaling, 1:1000) LC3B (Cell Signaling, 1:1000), and 1 h with a secondary antibody. Each membrane was then incubated with mouse monoclonal anti-β-actin (1:100,000, Santa Cruz Biotechnology, Inc., Bergheimer, Heidelberg, Germany ). The proteins were detected by chemiluminescence using ECL Western blotting (Amersham, GE Healthcare Life Science, Chicago, IL, USA). The signals were detected by a digital scanner and quantified with Image Lab Software (Bio-Rad Laboratories, Inc., Hercules, CA, USA). For cyclin-dependent kinase CDK4, p21, p53, SQSTM1/p62, LC3B and NF-κB p65 analysis, cells were plated at a density of 0.5 × 10^6^ cells in DMEM with 0.5% serum for 48 h. The inductions were performed every 24 h with GS 1 mg/mL and DMSO 0.3% as vehicle control and the cells collected after 24, 48, and 72 h of treatment. For pospho-mTOR (Ser2448) (Cell Signaling, 1:1000), mTOR (Cell Signaling, 1:1000), pospho-p70 S6 kinase (Thr389) (Cell Signaling, 1:1000), and p70 (Cell Signaling), treatment with GS 1 mg/mL was performed at 15 min, 30 min, 1 h, 2 h, 4 h. DMSO 0.3% was used as vehicle control. The membranes were incubated overnight with anti-pospho-mTOR (Ser2448) and pospho-p70 S6 kinase (Thr389) in 2.5% bovine serum albumin (BSA). The membranes were stripped to be re-probed overnight with anti- mTOR and -p70 S6 kinase antibodies.

### 2.8. Western Blot Analysis of Apoptotic Pathway in U87Mg Cells Treated with GS Extract

The potential apoptotic effect of GS 1 mg/mL were assessed by Western blot analysis of caspases 3 and 9 and Poly (ADP-ribose) polymerase (PARP). Samples were prepared as previously reported for cell cycle proteins analysis and PVDF membranes were incubated with caspase 3 (Cell Signaling, 1:1000), caspase 9 (Cell Signaling, 1:1000) and PARP (Cell signaling 1:1000) in 2.5% milk in TBS-T overnight at 4 °C.

### 2.9. DNA Ladder

U87Mg cells were plated and cultured as reported in Section 2.7. Treatment with 1 mg/mL GS extract was performed every 24 h and cells were collected at 24 and 72 h of exposure. DMSO 0.3% was used as control vehicle. DNA was extracted by QIAamp DNA mini kit (QIAGEN, Strasse 1, Hilden, Germany), and analysis of DNA fragmentation observed by electrophoresis on 2% agarose gel.

### 2.10. Immunofluorescence Detection of Cytoskeletal Proteins in GS-Treated U87Mg Cells

U87Mg cells (5 × 10^3^) were plated in 8-well chamber slides in DMEM with 0.5% serum for 48 h. Cells were treated with GS 1 mg/mL in DMEM with 10% FBS for 24 h and the morphological change induced by GS treatment, assessed by immunofluorescence staining for microtubule-associated protein α-tubulin and specific glioma marker vimentin. DMSO 0.3% was used as vehicle control. In detail, U87Mg cells were fixed with 4% formalin (Diapath S.p.A., Martinego, BG, Italy) for 20 min and permeabilised with 0.1% Triton (Invitrogen, Waltham, MA, USA) for 30 min. After blocking with 10% specific serum, the cells were incubated with anti-vimentin (prediluted; Roche diagnostic) and anti-α tubulin (Abcam, 1:200) overnight at 4 °C. After washing with 0.025% PBS-Tween-20, cells were incubated with secondary antibody anti-mouse fluorescein (1:100; Vector Laboratories) and anti-rabbit Cy3 (Merck Millipore, Darmstadt, Germany, 1:200) in 2% serum for 1 h at room temperature. The slides were counterstained with DAPI Mounting Medium (Vectashield, Burlingane, CA, USA) for nuclei detection and analysed with a fluorescence microscope at 20× and 40× magnification.

### 2.11. Immunofluorescence Detection of LC3-Puncta under Bafilomycin Treatment

Autophagic activity was evaluated starving U87Mg cells in Hank’s balanced salt solution (HBSS, Gibco) and pretreating them with Bafilomycin A1 100 nM (Bioviotica Naturstoffe GmbH, Dransfeld, Germany). In detail, U87Mg cells (5 × 10^3^) were plated in 8-well chamber slides in DMEM with 0.5% serum for 48 h. For the LC3 puncta detection, GBM cells were starved in HBSS and pretreated with Bafilomycin A1 100 nM for 4 h. After removal of Bafilomycin A1, GS extract (1 mg/mL) was added in HBSS and complete medium for 12 h. Cells that were cultured, respectively, in complete medium and HBSS and that were treated with 0.3% DMSO as vehicle, served as controls. For immunofluorescence detection of LC3 puncta, protocol mentioned in Section 2.10 was applied, using anti-LC3B antibody (Cell Signaling, 1:200).

### 2.12. Statistical Analysis

Experiments were performed in triplicate and data were expressed as mean ± SEM. Statistical significance was determined by Student’s *t*-test, one-way or two-way ANOVA, considering *p*-value < 0.05 as statistically significant.

## 3. Results

### 3.1. Effects of GS Extract on Glioblastoma Cells U87Mg

**Figure 1:**
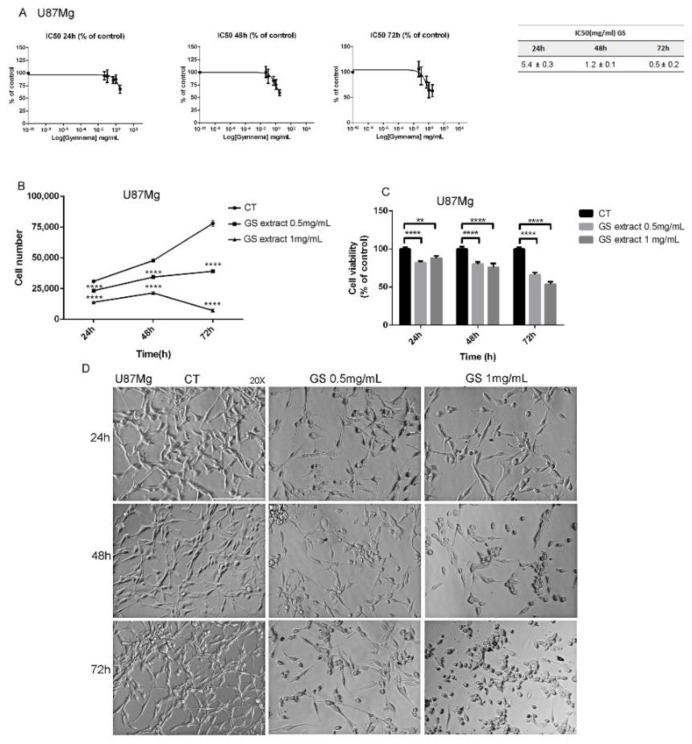
(**A**) IC50-values of U87Mg cells after 24, 48, and 72 h of incubation with GS extract 0.05, 0.1, 0.5, 1 and 3 mg/mL and DMSO 0.3% as vehicle control, respectively. (**B**) Time- and dose-dependent growth inhibition of GS-treated U87Mg cells. DMSO 0.3% was used as vehicle control. (**C**) Cytotoxic effect of GS 0.5 and 1 mg/mL at 24, 48, and 72 h post-treatment on U87Mg cell line. DMSO 0.3% was used as vehicle control. (**D**) Morphological changes of U87Mg human glioblastoma cells after 24, 48, and 72 h of exposure to GS 0.5 and 1 mg/mL. DMSO 0.3% was used as vehicle control. Magnification 20×. For all the experiments, values are the means ± SEM of 3 individual determinations. Unpaired *t*-test, *p*-value < 0.05. According to GraphPad Prism, ** *p*-value 0.001 to 0.01 (very significant), **** *p*-value < 0.0001 (Extremely significant).

The cytotoxic effects of GS extract were evaluated by IC50 values at 24, 48, and 72 h using the continuous GBM U87Mg cell line. As evidenced in Figure 1A, the IC50 values significantly decreased from 5.4 ± 0.3 mg/mL at 24 h to 0.5 ± 0.2 mg/mL at 72 h. Considering the high cytotoxic effects, GS extract was daily administered at concentrations of 0.5 and 1 mg/mL at different time intervals (24, 48, and 72 h). Cell count revealed a statistically significant time- and dose-dependent reduction in cell growth (Figure 1B), with more than 50% and 90% of growth inhibition rate at 72 h of treatment, respectively, with GS 0.5 and 1 mg/mL. Similarly, GS extract interfered with cellular metabolic activity as reported in Figure 1C. GS extract induced a remarkable decrease in U87Mg cell viability in a dose- and time-dependent manner. A significant viability reduction of 15% was observed already after 24 h of exposure to GS extract 0.5 and 1 mg/mL. This increased reduction was remarkable at 48 h, where it reached 20% and 24%, respectively.

After 72 h, cell viability inhibition was 30% and 45%, respectively. Furthermore, GS extract induced critical morphological changes of U87Mg cells, microscopically visible as round-shaped with a loss of filaments and cell shrinkage (Figure 1D).

### 3.2. Clonogenic Survival and Motility Capacity of GBM Cells after GS Extract Treatment through Potentially Inhibition of NF-κB

**Figure 2:**
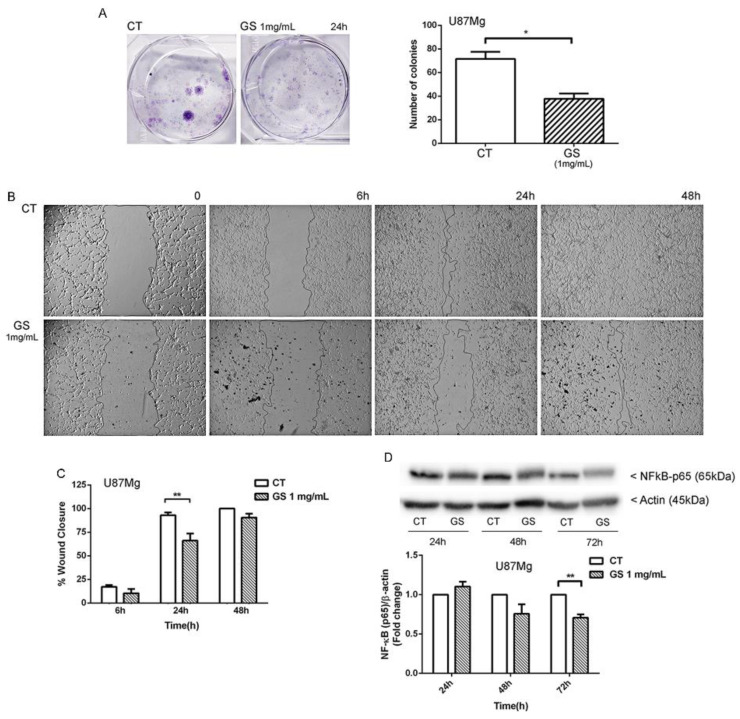
(**A**) Colony formation after 24 h treatment of GS extract 1 mg/mL. DMSO 0.3% was used as vehicle control. (**B**) Wound healing assay of U87Mg at T0 and 6, 24, 48 h post-scratch in control (U87Mg cells treated with DMSO 0.3%) and cells treated with GS 1 mg/mL and (**C**) quantification of the cell-free area at corresponding time points. Data were analysed with two-way ANOVA test and significance reported in accordance with GraphPad Prism, ** *p*-value 0.001 to 0.01 (very significant). (**D**) Western blot analysis of NF-κB, respectively, under GS 1 mg/mL treatment and DMSO 0.3% as vehicle control, and normalisation with housekeeping gene Actin. Densitometric analysis of protein levels represent the means ± SEM of 3 individual determinations. Data are expressed as fold change over control-treated cells. * Unpaired *t*-test. According to GraphPad Prism, * *p*-value 0.01 to 0.05 (significant), ** *p*-value 0.001 to 0.01 (very significant).

The cytostatic effect of GS extract was further investigated with colony-forming assay, which revealed that the number of colonies was reduced by about 47% in GS-treated GBM cells compared with control cells (Figure 2A). GS extract also interfered with cell motility. GS-treated cells closed the scratch more slowly than untreated cells, with an average percentage of closure of the scratch area of about 66% compared with 92% of control cells (Figure 2B,C). Considering the role of NF-κB transcription factor in the tumour-invasion capacity of GBM cells [15], the expression level of this protein was investigated. Not surprisingly, NF-κB p65 protein expression decreased after 72 h of treatment with 1 mg/mL GS extract (Figure 2D).

### 3.3. Dynamic Rearrangement of Cytoskeletal Proteins in GS-Treated U87Mg Cells

**Figure 3:**
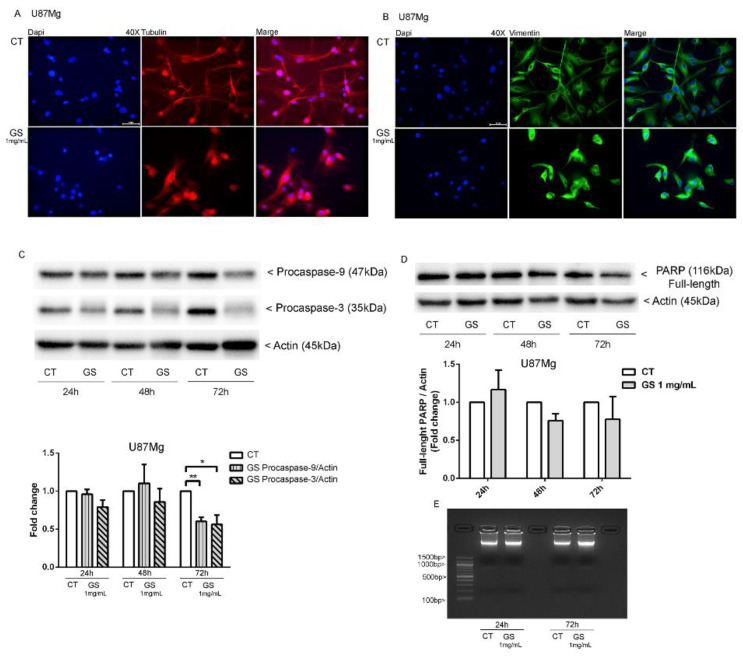
Reorganisation of cytoskeletal proteins in 1 mg/mL GS-treated cells. DMSO 0.3% was used as vehicle control. Immunofluorescence for α-tubulin (**A**) and vimentin (**B**). (**C**) Western blot analysis of apoptotic-associated proteins Procaspase 9, Procaspase 3, and (**D**) PARP in U87Mg cells treated with GS 1 mg/mL and DMSO 0.3% as vehicle control. Densitometric analysis of protein levels represent the means ± SEM of 3 individual determinations. Data were normalised to housekeeping gene Actin and are expressed as fold change over control-treated cells. * Unpaired *t*-test. According to GraphPad Prism, * *p*-value 0.01 to 0.05 (significant), ** *p*-value 0.001 to 0.01 (very significant). (**E**) Agarose gel for DNA ladder at 24 and 72 h from treatment of U87Mg with GS 1 mg/mL. DMSO 0.3% was used as vehicle control. There is no evidence of DNA fragmentation.

Morphological changes of GBM cells are linked to the activation of cell death pathway [16], implying a dynamic rearrangement of cytoskeletal proteins. Immunofluorescence staining for microtubule-associated protein α-tubulin and intermediate filament vimentin evidenced a relevant reorganisation of cytoskeletal proteins in GS-treated cells. GS treatment induced the depolymerisation of microtubules, with α-tubulin localised around the nucleus rather than distributed in the cytoplasm, as in untreated cells (Figure 3A). The distribution of vimentin, a marker of glioblastoma, also changed in cells treated with GS extract, with a distribution pattern similar to α-tubulin (Figure 3B). Furtherly, we analysed the involvement of apoptotic pathways. In the Western blot shown in Figure 3C, long-term treatment of U87Mg cells with 1 mg/mL GS seemed to trigger slight activation of apoptotic markers only after 72 h of exposure. The results obtained showed only reduction in procaspase 3 and 9. Neither the presence of active caspases, nor PARP activation nor DNA fragmentation was evident. These negative results led us to exclude apoptosis as a cell death mechanism in GS-treated U87Mg cells.

### 3.4. Analysis of Autophagy Markers in GS-Induced Cytotoxicity of Glioblastoma Cells U87Mg

**Figure 4:**
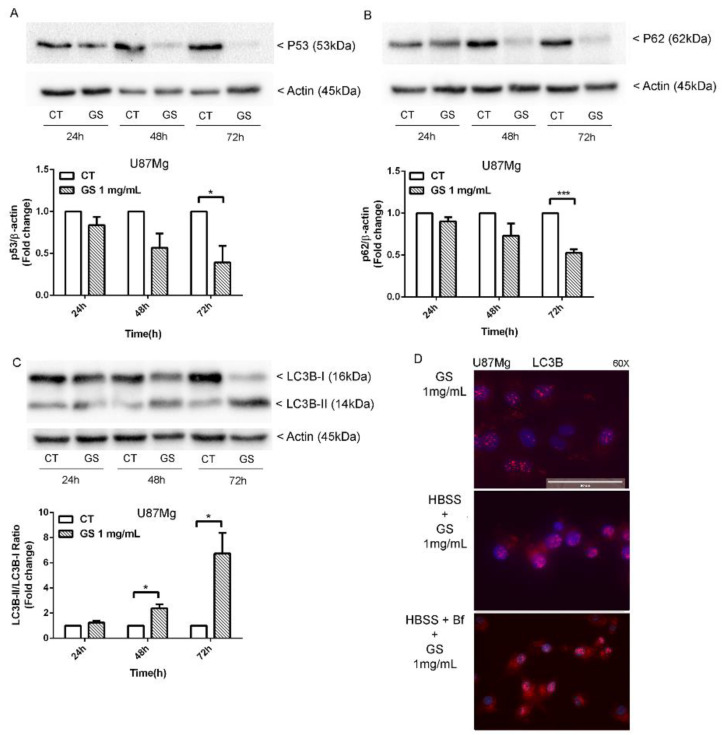
Autophagy modulation by GS extract. Western blot of U87Mg treated, respectively, with GS 1 mg/mL and DMSO 0.3% as vehicle control for 24, 48, and 72 h showed a reduction in p53 (**A**) and P62 (**B**) protein levels, and a decrease in LC3B- I with an increase in active form LC3B-II (**C**). Densitometric analysis of protein levels represent the means ± SEM of 3 individual determinations. Data were normalised to housekeeping gene Actin and are expressed as fold change over control-treated cells. * Unpaired *t*-test. According to GraphPad Prism, * *p*-value 0.01 to 0.05 (significant), *** *p*-value 0.0001 to 0.001 (Extremely significant). (**D**) Immunofluorescence of LC3B puncta, which were more evident after autophagy blocking with Bafilomycin A1 (Bf). Magnification 60× with Evos FL microscope.

Western blot analysis of proteins involved in autophagic pathway was performed. Since p53 inhibition is critical in the activation of autophagy [17,18], p53 expression was evaluated by Western blot analysis, which revealed a strong reduction in this protein after 72 h of exposure to GS extract (Figure 4A). Alongside this, the expression of autophagy-associated protein p62 showed more than 50% of reduction (Figure 4B), while at least 6-fold increase in LC3B-II/LC3B-I ratio (Figure 4C) was observed. These results indicated that GS extract could induce the autophagosomes formation, as confirmed by immunofluorescence staining for LC3B. A remarkable accumulation of LC3B puncta was visible in U87Mg cells pre-treated for 4 h with 100 nM Bafilomycin A1, the inhibitor of autophagosome and lysosome fusion, and 1 mg/mL GS extract in HBSS medium for 16 h, compared with control cells cultured in HBSS or complete medium with 1 mg/mL GS extract (Figure 4D).

**Figure 5:**
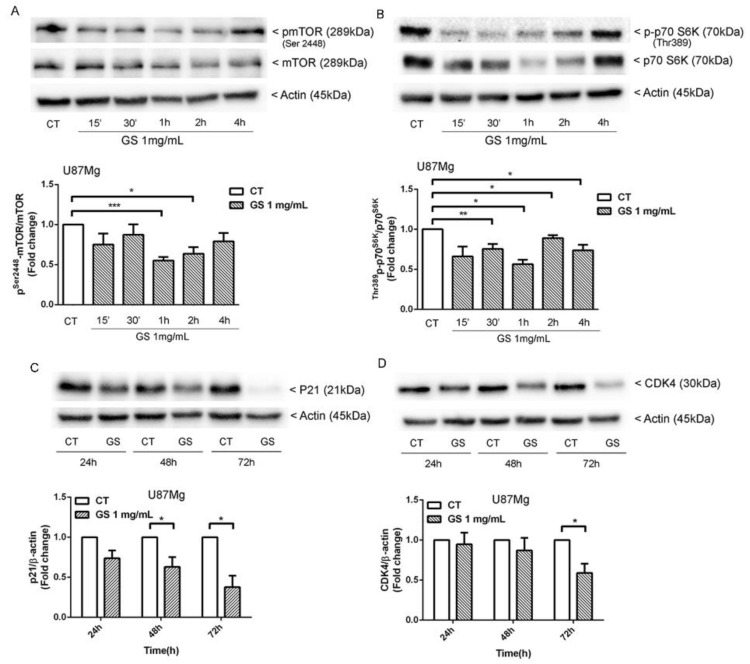
(**A**) In U87Mg cells, GS extract 1 mg/mL induced a significant reduction in the pospho^Ser2448^-mTOR/mTOR and (**B**) p70-^Thr389^S6K/p70-S6K ratio compared with cells treated with DMSO 0.3% as vehicle control. Western blot analysis of (**C**) p21^WAF1/CIP1^ and (**D**) CDK4 expression after treatment of U87Mg, respectively, with GS 1 mg/mL and DMSO 0.3% as vehicle control. Densitometric analysis of protein levels represent the means ± SEM of 3 individual determinations. Data were normalised to housekeeping gene Actin and are expressed as fold change over control-treated cells. * Unpaired *t*-test. According to GraphPad Prism, * *p*-value 0.01 to 0.05 (significant), ** *p*-value 0.001 to 0.01 (very significant), *** *p*-value 0.0001 to 0.001 (Extremely significant).

On the basis of these findings, the upstream effectors of autophagy were investigated. The molecular target of rapamycin (mTOR) has been reported to be upregulated in glioblastoma, where it controls cell growth rate, stem cell proliferation, and GBM recurrence. GS extract induced a significant reduction in the pospho^Ser2448^-mTOR/mTOR ratio in short-term treatment of U87Mg (Figure 5A), similarly to mTOR inhibitor Rapamycin. Therefore, inactive mTOR did not phosphorylate its downstream target p70-^Thr389^S6K as shown in Figure 5B, where the ratio p70-^Thr389^S6K/p70-S6K decreased reaching a maximum reduction after 1 h of treatment, according to the maximum inhibition of mTOR activity. A decrease in total protein p70-S6K was also detected. This event was already observed in K562 cells treated with Rapamycin, doxorubicin, and a combination of both drugs [19]

### 3.5. Inhbition of p21^WAF1/CIP1^ Expression and CDK4-Induced Macroautophagy in GS-Treated Glioblastoma Cells U87Mg

Inhibition of mTOR by rapamycin and its derivative RAD001 reduced the level of total p21^WAF1/CIP1^ [20]. In this regard, considering the GS-induced inhibition of mTOR, the effect of GS extract on p21 expression in U87Mg cells was investigated. The results revealed a strong reduction in p21 at 48 h (37%) and (62%) at 72 h of exposure to GS extract (Figure 5C). Since CDK4/6 inhibition by chemical inhibitors or siRNA has recently been reported to induce macroautophagy in different cancer cells [21,22], CDK4 expression has also been analysed, revealing a significant decrease at 72 h of treatment with this natural extract, with at least 40% reduction compared with control cells (Figure 5D).

## 4. Discussion

Therapeutic failure and poor prognosis of GBM patients is due to the development of resistance and considerable side effects of conventional therapy. Current research efforts are focusing on the use of natural substances as adjuvants to traditional therapy for GBM treatment for their potential beneficial effects in terms of survival, modulation of the immune system and patients’ quality of life [23,24].

In this study the anti-proliferative effects of the natural compound GS on human U87Mg glioblastoma cells were evaluated. The IC50-values of the GS extract evaluated on U87Mg cells at 24, 48, and 72 h, respectively, showed a time-dependent cytotoxic effect.

The effects of GS extract on cell growth were evaluated by growth curves, by daily treating U87Mg cells with GS 0.5 and 1 mg/mL according to the IC50-values. The plot evidenced a strong growth reduction already after 24 h of treatment in a time- and dose-dependent manner, as confirmed by subsequent cell viability and clonogenic assay experiments. From the latter in particular, a 47% reduction in clonogenic potential was observed in U87Mg cells treated with GS 1 mg/mL for 24 h. Moreover, cell migration, a typical feature of cancer cells as index of invasiveness and malignancy [25], was investigated by wound-healing assay. Not surprisingly, GS extract negatively interfered with U87Mg cells motility and migration, and it was linked to the significant inhibition of NF-κB transcription factor (Figure 2), which might impair the invasive phenotype of glioblastoma, as previously reported [15].

A deep rearrangement of cytoskeletal proteins α-tubulin and vimentin was also observed by immunofluorescence staining of U87Mg treated with GS extract, already after 48 h, started to lose the typical polygonal shape by assuming a rounded shape. These features led us to further investigate the involvement of cell death pathways in GS-induced cytotoxicity. Western blot analysis showed, at 72 h of treatment, a reduction in procaspase 9 expression followed by decrease in its downstream target procaspase 3. The subsequent analysis of another apoptotic marker, PARP, revealed that the level of this protein did not change, and the absence of its cleaved form confirmed that apoptosis was not involved. The above assumption was also confirmed by the lack of DNA fragmentation (Figure 3).

These findings are in line with our previous study, in which we demonstrated that rapamycin-induced cell death of glioblastoma cells was not associated with apoptosis, but with a massive activation of the complex autophagic system [8].

Type II programmed cell death or autophagy maintains cellular homeostasis and is dynamically activated under stressful conditions. These events are supported by the activation in the network of relevant proteins known to trigger and regulate autophagy and by their rapid and reversible post-translational modifications such as phosphorylation [26]. p53 protein represents a sorting key of signals towards the apoptotic or autophagic pathways. Several studies explained that knockout, knockdown, or pharmacological inhibition of p53 can induce autophagy in human, mouse, and nematode cells [17,18]. Our results are consistent with these studies. The downregulation of p53 could be related to the activation of autophagic pathway in U87Mg cells treated with GS extract.

A further key player of autophagy control in GBMis p62, which acts as an oncogene [27]. Therefore, it has been proposed as a new potential therapeutic target for gliomas. According to this evidence, in our study a relevant reduction in p62 expression was detected in long-term treatment with GS extract. A powerful increase in LC3 II/I ratio, in the same experimental conditions, confirmed a massive activation of autophagy.

The maturation of autophagosomes, hallmark feature of autophagy, was also investigated by immunofluorescence staining for LC3B under pre-treatment of U87Mg cells with Bafilomycin A1 in HBSS medium. Inhibition of autophagosome and lysosome fusion by Bafilomycin A1 prevents the formation of autophagolysosome. In these conditions, LC3-positive puncta dramatically increased in U87Mg cells pretreated with Bafilomycin A1 for 4 h while LC3B puncta were not evident in control cells.

The mechanism of action of GS extract is comparable with the standard type 2 antidiabetic drug Metformin, which exhibited anticancer properties inducing autophagy via AMPK-mTOR signaling pathway [28]. GS extract, as well as Metformin, reduced the phosphorylation levels of mTOR and, as consequence, of its downstream target P70 S6 kinase, in short-term treatment of U87Mg cells.

Finally, the prominent reduction in p21^WAF1/CIP1^, similarly to rapamycin and its derivative RAD001 [20], as well as CDK4 expression [21,22], supported the role of autophagy in GS-induced cytotoxicity.

## 5. Conclusions

In conclusion, we propose GS extract as an autophagy inducer in glioblastoma cells U87Mg. The use of natural substances that act in synergy with the traditional drugs for the treatment of glioblastoma could open the door to new treatment options for this disease with extremely poor prognosis. The goal of this research was to evaluate the biological effects of GS on glioblastoma cells with the aim to restore cell death mechanisms. Further investigations will be needed to introduce GS extract as an adjuvant to the traditional chemoradiotherapy treatment of glioblastoma.

## Data Availability

The data presented in this study are available on request from the corresponding author.
